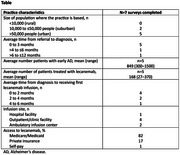# Real‐World Insights on the Lecanemab Patient Pathway in Early Alzheimer’s Disease in the United States

**DOI:** 10.1002/alz70861_108606

**Published:** 2025-12-23

**Authors:** Michael Henry Rosenbloom, Marwan N. Sabbagh, Jose Soria‐Lopez, Gregory Cooper, Samuel Giles, Cara Leahy, Martin Sadowski, Curtis Schreiber, Paul E Schulz, David C Weisman, Christian J Camargo, Brooke Allen, Courtney Adams, Daryl Jones

**Affiliations:** ^1^ University of Washington Memory and Brain Wellness Center, Seattle, WA USA; ^2^ Barrow Neurological Institute, Phoenix, AZ USA; ^3^ University of California San Diego, La Jolla, CA USA; ^4^ The Neuron Clinic, San Diego, CA USA; ^5^ Norton Neuroscience Institute, Louisville, KY USA; ^6^ Memory Treatment Centers, Jacksonville Beach, FL USA; ^7^ Memorial Healthcare Institute for Neuroscience, Owosso, MI USA; ^8^ New York University Langone Health, New York, NY USA; ^9^ Missouri Memory Center, Citizens Memorial Hospital, Bolivar, MO USA; ^10^ John P. and Kathrine G. McGovern Medical School at UTHealth, Houston, TX USA; ^11^ Abington Neurologic Associates, Abington, PA USA; ^12^ University of Miami Miller School of Medicine, Miami, FL USA; ^13^ Roaring Fork Neurology, Basalt, CO USA; ^14^ Eisai Inc, Nutley, NJ USA; ^15^ Eisai Inc., Nutley, NJ USA

## Abstract

**Background:**

Lecanemab‐irmb (LEQEMBI®) is indicated for the treatment of patients with Alzheimer’s disease (AD) in the mild cognitive impairment or mild dementia stage. The optimized patient pathway enables early and accurate diagnosis of AD and supports patients and care partners through initiation of treatment and appropriate monitoring. This analysis described the lecanemab patient pathway across different medical centers and/or healthcare facilities in the United States.

**Method:**

This multicenter, retrospective case series and patient pathway study was conducted in 15 geographically diverse neurology clinics, each abstracting deidentified medical chart data for up to 25 patients receiving lecanemab (≥7 infusions) and 1 neurologist per site completing an electronic survey plus an interview. Data collected included sociodemographic characteristics, clinical characteristics, AD diagnosis, and lecanemab use, in addition to practice characteristics and norms to assess best practices. This interim analysis (cutoff date: April 11, 2025) includes ∼25% of the expected total study population (final data cut: May 23, 2025). The protocol received central institutional review board exemption.

**Result:**

This is an interim analysis of 7 surveys out of a possible 15 surveys completed by neurologists. Practice characteristics for the centers surveyed are illustrated in the Table. On average, 168 patients are treated with lecanemab at each center. They are mostly diagnosed using CSF or amyloid PET, and APOE testing is performed either at the initial consultation (*n* =3) or after amyloid pathology is confirmed (*n* =3). Most patients treated with lecanemab are over 65 years old (81%), access their infusions within an outpatient/clinical facility, and receive their first infusion within 4 months of diagnosis. Common cognitive assessments used include Mini‐Mental State Examination (MMSE; n=5) and the Montreal Cognitive Assessment (MoCA; n=6). Clinical assessments are generally conducted in person every 6 months.

**Conclusion:**

This interim analysis indicates a relatively consistent application of diagnostic and treatment protocols across the lecanemab patient pathway. In the full data set analysis (data cutoff: May 23, 2025), practice characteristics will be further reviewed through interviews to identify needs and opportunities that could guide initiatives aimed at supporting the real‐world integration of lecanemab into standard clinical care.